# General practitioner and patient perspectives on intranasal corticosteroids for allergic rhinitis: Treatment duration and obstacles to adherence, findings from a recent survey^☆^

**DOI:** 10.1016/j.waojou.2024.100925

**Published:** 2024-06-25

**Authors:** Désirée E.S. Larenas-Linnemann, Pornanan Domthong, Renata C. Di Francesco, Ruperto González-Pérez, Manish Verma

**Affiliations:** a Centro de Excelencia en Asma y Alergia, Hospital Médica Sur, Mexico City, Mexico; b Internal Medicine, Khon Kaen Hospital, Khon Kaen, Thailand; c Hospital das Clínicas da Faculdade de Medicina da Universidade de São Paulo, São Paulo, Brazil; d Allergy Department and Severe Asthma Unit, Hospital Universitario de Canarias, Santa Cruz de Tenerife, Spain; e GSK, Mumbai, India

**Keywords:** Allergic rhinitis, Physician questionnaire, Patient questionnaire, Adherence, Intranasal corticosteroid

## Abstract

**Background and objective:**

Currently, there are no guideline recommendations for the duration of intranasal corticosteroid (INCS) treatment for allergic rhinitis (AR). We aimed to catalogue real-world AR-INCS prescription patterns.

**Materials and methods:**

This multicenter, non-interventional, cross-sectional study used online general practitioner (GP) and patient surveys from 4 countries. Eligible GPs had 3–35 years of practical experience, regularly prescribed INCSs for AR treatment, and had managed ≥5 patients with AR per month according to Allergic Rhinitis and its Impact on Asthma (ARIA) guidelines in the previous year. Eligible patients with AR were non-pregnant females or males, aged 18–65 years, previous AR-INCS users (≥12 months), and receiving GP-prescribed AR therapy. Survey participants were from countries with 15–50% AR prevalence and mostly prescription-only INCS use of ≥100 million units annually (Brazil, Mexico, Spain, Thailand). GP surveys and GP-completed patient record forms (PRFs) gathered AR-care and INCS-use data over 10 months; each GP completed patient record forms (PRFs) for 3 patients with AR under their care. The patient survey reflected actual AR-INCS experience, treatment duration, and adherence factors from patient perspectives. The target sample size was 75 GPs, 75 patients, and ≥30 respondents per country.

**Results:**

From 900 GP-PRFs, the mean GP-recommended AR-INCS durations reported were 8.4 (Brazil), 8.3 (Mexico), 5.4 (Spain), and 6.4 (Thailand) weeks. From 300 patient surveys, mean reported INCS recommended durations were 6.4 (Brazil), 5.1 (Mexico), 4.0 (Spain), and 4.9 (Thailand) weeks; reported actual use durations were 6.2, 4.8, 3.6, and 6.4 weeks, respectively. The most frequent GP-PRF-reported factors influencing AR-INCS treatment duration were symptom severity (76–85%), symptom recurrence (49–73%), and existing comorbidities (33–57%). The most frequent GP-PRF-reported obstacles to adherence included forgetting to take medication regularly (54–100%), subsiding symptoms (42–91%), and being unable to continue activities (33–51%). Subsiding symptoms (36–53%) and reaching the prescription duration end (20–51%) were most frequent obstacles reported by the patient survey. Patients from all surveyed countries indicated that they visited the GP, a different physician, or a pharmacy for assistance with symptom recurrence; some patients also self-medicated.

**Conclusions:**

Real-world AR-INCS prescription durations vary between countries and actual use tends to be shorter than prescribed. Understanding underlying factors may support appropriate AR-INCS use. The study was not powered to statistically compare intercountry differences; hence, comparisons have not been drawn, and the small sample may not reflect a complete picture of clinical practice and patients with AR in each country.

## Introduction

Allergic rhinitis (AR), the most common type of chronic rhinitis, is characterized by nasal congestion, itch, rhinorrhoea, sneezing, and anosmia in patients with severe obstruction.^1^ The current Allergic Rhinitis and its Impact on Asthma (ARIA) guidelines^2^ categorize the duration of AR into intermittent or persistent patterns, and the severity as mild or moderate-to-severe; however, the ARIA guidelines do not stipulate a specific duration of intranasal corticosteroid (INCS) treatment. Similarly, the European Forum for Research and Education in Allergy and Airway Diseases (EUFOREA) proposed treatment algorithm for AR recommends INCSs as one of the first-line options for patients presenting with at least 2 nasal symptoms suggestive of AR but provides no specific guidance on treatment duration.^3^^,^^4^ The existing recommendations simply state that treatment should be continuous for persistent cases, or should cover the period of affliction for intermittent cases. Treatment should also depend on symptom severity, with longer treatment durations prescribed for patients with more severe symptoms, which are associated with a higher degree of allergic inflammation.^5^^,^^6^

There is a disconnect between INCS prescriptions for AR and INCS use by patients in real-world settings. Most healthcare providers, including allergists and general practitioners (GPs), prescribe medications for the entire season and recommend that patients use them regularly, even on days when they experience few symptoms.^7^ In contrast, most patients use their medications on demand when their AR is not well controlled,^2^^,^^8^ which fails to confer maximal treatment benefit; persistent inflammation of the nasal mucosa remains despite minimal symptoms.^9^^,^^10^

We aimed to catalogue real-world INCS prescription patterns for AR. The primary objective was to determine the prescribed treatment duration of INCS in Brazil, Mexico, Spain, and Thailand as reported by GPs through patient record forms (PRFs) for specific patients under their care. We captured 2 different perspectives, from GPs and patients, on reasons for non-adherence to prescribed INCS duration. GP-reported reasons for recommending INCS use in patients with AR and factors impacting the prescribed treatment duration were also gathered, alongside any patient-reported durations of INCS prescriptions from GPs versus actual INCS use.

## Methods

### Objectives

The primary objective for this study was to determine the GP-prescribed INCS treatment duration in patients with AR, as collected through GP-completed PRFs in the participating countries (Brazil, Mexico, Spain, and Thailand). Prescribed treatment durations were reported via GP-completed PRFs, by GPs prescribing for specific patients under their care.

Secondary objectives (detailed below) were explored using GP-PRFs as well as GP-completed and patient-completed online surveys. GP and patient surveys provided information from their own recall.

PRFs aimed to determine reasons for recommending INCS use in patients with AR, factors impacting the prescribed treatment durations, and GP-reported reasons for patient non-adherence to prescribed treatment durations. GP surveys aimed to determine reasons for recommending INCS use in patients with AR, factors impacting prescribed treatment durations, GP-reported reasons for patient non-adherence, and reasons for subsequent treatment decisions, based on patients’ responses to INCSs. Patient surveys (patients were not linked to the GPs participating in the GP survey) aimed to determine patient-reported prescribed INCS durations, the duration of GP-prescribed INCSs on their latest prescription (at their most recent visit within the last three months), the duration of actual INCS use, and reasons for non-adherence to or discontinuation from the prescribed treatment duration.

### Study design and ethics

**Fig. 1:**
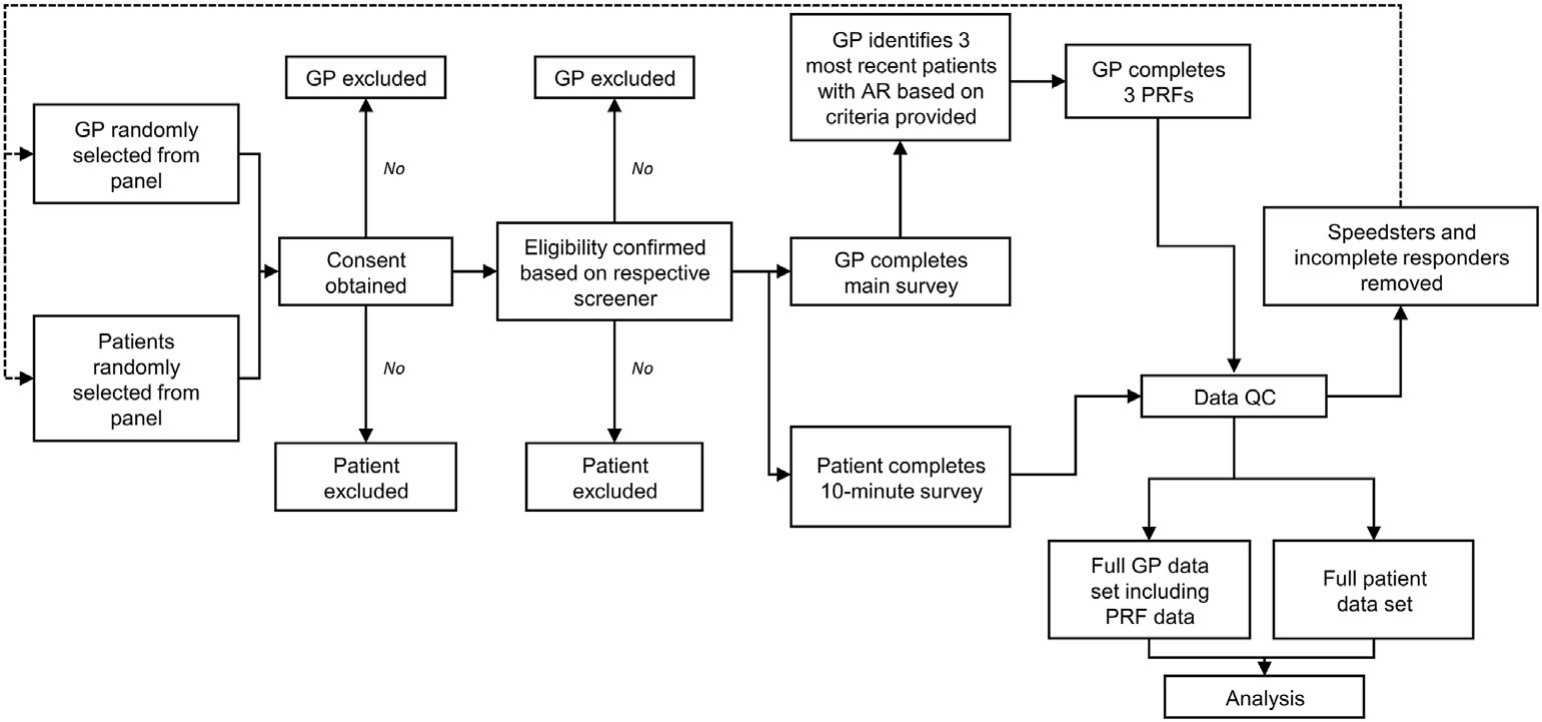
Study design. AR, allergic rhinitis; GP, general practitioner; PRF, patient record form; QC, quality check

Fig. 1 depicts the key steps in this non-interventional, cross-sectional study from initiation to analysis. Participating countries had 15–50% AR prevalence, INCS dispensation mainly by prescription, and INCS use of ≥100 million units annually. All participants (GPs and patients) were screened and enrolled if they met the inclusion criteria and gave their consent for data to be analysed and published anonymously.

Following consideration of the study documents (protocol, survey, and consent form), ethical approval exemption was granted according to FDA 21 CFR 56.104 and 5CFR46.104(d)(2): Tests, Surveys, Interviews. The study was also reviewed by the Institutional Review Board and by the Human Research Ethics Committee at Khon Kaen Hospital in Thailand.

### General practitioners

General practitioner (GP) surveys (Supplemental Appendix A) and GP-completed PRFs (Supplemental Appendix B) collected perspectives on AR care and INCS use over 10 months; each GP completed a survey in addition to 3 PRFs related to 3 of their patients with AR. GP surveys were conducted from January to March 2023 in Brazil, Mexico, and Spain, and from March to May 2023 in Thailand.

GPs were eligible for study participation if they had 5–35 years of experience in practice, spent ≥60% of their time in direct patient care, had personally seen and treated patients diagnosed with AR within the last 3 years, regularly prescribed INCSs to patients diagnosed with AR, had treated ≥5 patients with AR on average per month in the previous year according to ARIA guidelines, and were able to fill out 3 PRFs for patients with AR. GPs were ineligible if they reported any reason for being unable to complete the study.

### Patients

A separate patient survey (Supplemental Appendix C) collected data on INCS use for AR therapy, including treatment experiences and factors influencing adherence and discontinuation. Patient surveys were conducted from December 2022 to January 2023 in Brazil, Mexico, and Spain, and from March to April 2023 in Thailand.

Patients with AR were eligible if they were aged 18–65 years at the time of consent, received INCSs to treat AR for ≥12 months, and were prescribed AR treatment by a GP who did not participate in this study. Exclusion criteria were pregnancy or planned pregnancy, lactation, and reporting any reason for being unable to complete the study.

### Study recruitment

GPs and patients were recruited via physician and patient panels (internal and external to the IQVIA network) from the participating countries. For GPs, participants were recruited from a panel of 108,000 respondents in Brazil, 15,000 in Spain, 8000 in Mexico, and 3000 in Thailand. Patients were recruited from IQVIA databases, with estimated panel sizes of 5000 in Thailand and 30,000 each in Brazil, Mexico, and Spain.

### Analyses

The target sample size was 75 GPs and 75 patients per country totalling 300 of each, and ≥30 respondents, with estimated response rates of 50% for GPs and 30–35% for patients. The sample size of 75 GPs per country was based on a two-sided 95% confidence interval and an expected GP-reported average prescription duration of 4 weeks (with a standard deviation of ±2 weeks) based on the opinions and experiences of subject matter experts. Based on these assumptions, the margin of error or distance of mean to limit was calculated as 0.46 weeks using PASS (NCSS, LLC, Version 19.0.1, East Kaysville, Utah, USA). The average GP-reported INCS prescription duration could have been thus reported with a margin of error of 0.46 weeks.

Data quality was assessed for every participant. Respondents who completed the survey in less than half of the average time expected and those providing inappropriate responses to open-ended questions were removed. Descriptive statistics, including the sample size (n) and mean, median and standard deviation were provided for continuous variables while frequencies and percentages were calculated for categorical and ordinal variables. Ranking of the categories was conducted to identify the most frequent responses for each survey question in each location. Data analysis was presented at the country level. No inter-country statistical analyses were conducted; however, noticeable trends are reported and discussed.

## Results

### General practitioners

The profile of participating GPs is noted in Table 1. GPs reported seeing 248–668 patients in a typical month (with the lowest number reported in Thailand and the highest in Spain). Of these patients, 15–29% were diagnosed with AR (the highest proportions were reported in Mexico and Brazil). The GP-reported number of patients with AR being managed for more than 1 year was lowest on average in Thailand (n = 25) and highest in Spain (n = 150). There was an almost equal split between mild and moderate-to-severe AR among patients receiving INCSs, with a slight skew towards mild AR in Spain and moderate-to-severe AR in Thailand.

**Table 1:** Profile of participating GPs.

Characteristic^a^	Brazil (N = 75)	Mexico (N = 75)	Spain (N = 75)	Thailand (N = 75)
Average cases per month (n)	396	311	668	248
Average AR diagnoses (n)	110	91	111	37
Average patients with AR treated (n)	69	68	118	19
Patients with:				
mild AR (%)	50	48	58	42
moderate-to-severe AR (%)	50	52	42	58

AR, allergic rhinitis; INCS, intranasal corticosteroid; GP, general practitioner.

a Patients treated with INCSs for more than one year

### Patients

Patient demographics and baseline characteristics are summarized in Table 2. Two-thirds of patients (67%) were 18–50 years old; the proportions of male and female patients were roughly equal in all countries except Thailand, where the majority (81%) were female. The majority (>67%) of patients were diagnosed >18 months prior to study enrolment; 75% experienced symptoms over multiple months and 25% reported symptoms for a few days or weeks during each episode.

**Table 2:** Demographics and baseline characteristics of participating patients.

Characteristic	Brazil (N = 75)	Mexico (N = 75)	Spain (N = 75)	Thailand (N = 75)
**Age, years (%)**				
18–65	40	47	37	51
36–50	45	45	36	48
51–65	15	8	27	1
**Sex (%)**				
Female	55	48	51	81
Male	45	52	49	19
**Occupation (%)**				
Unemployed	3	4	15	0
Retired	4	0	5	0
Self-employed	15	1	4	5
Part-time	5	7	12	0
Full-time	73	88	64	95
**Time since AR diagnosis (%)**				
12–18 months ago	28	33	25	16
>18 months ago	72	67	75	84
**Symptom frequency (%)**				
Daily or months at a time	75	75	75	76
A few weeks at a time	25	25	25	24

AR, allergic rhinitis

### GP-PRF findings

**Fig. 2:**
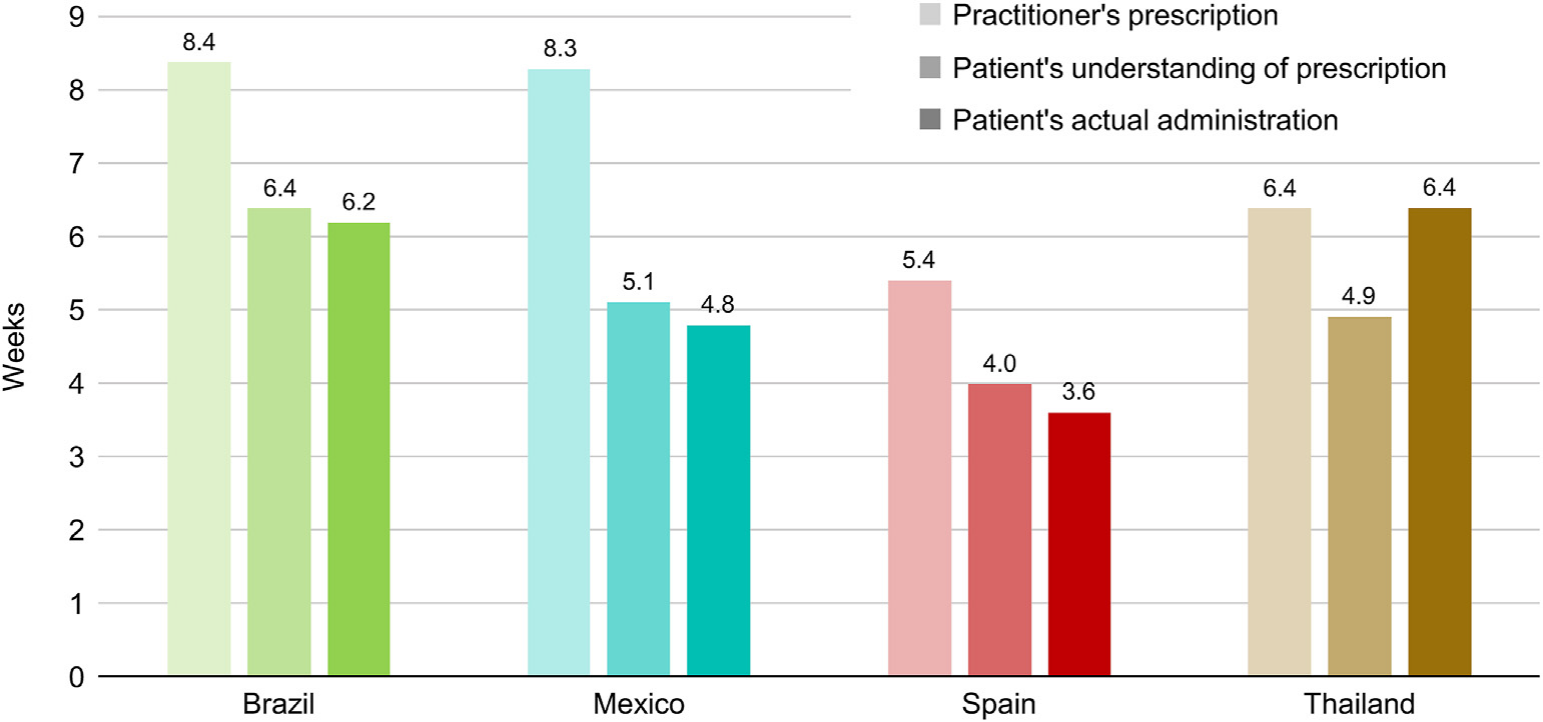
Duration (weeks) of INCS recommended use according to GP-PRFs (N = 300) and patient survey (N = 300), and duration of use of INCS according to patient survey. GP, general practitioner; INCS, intranasal corticosteroid; PRF, patient record form

**Fig. 3:**
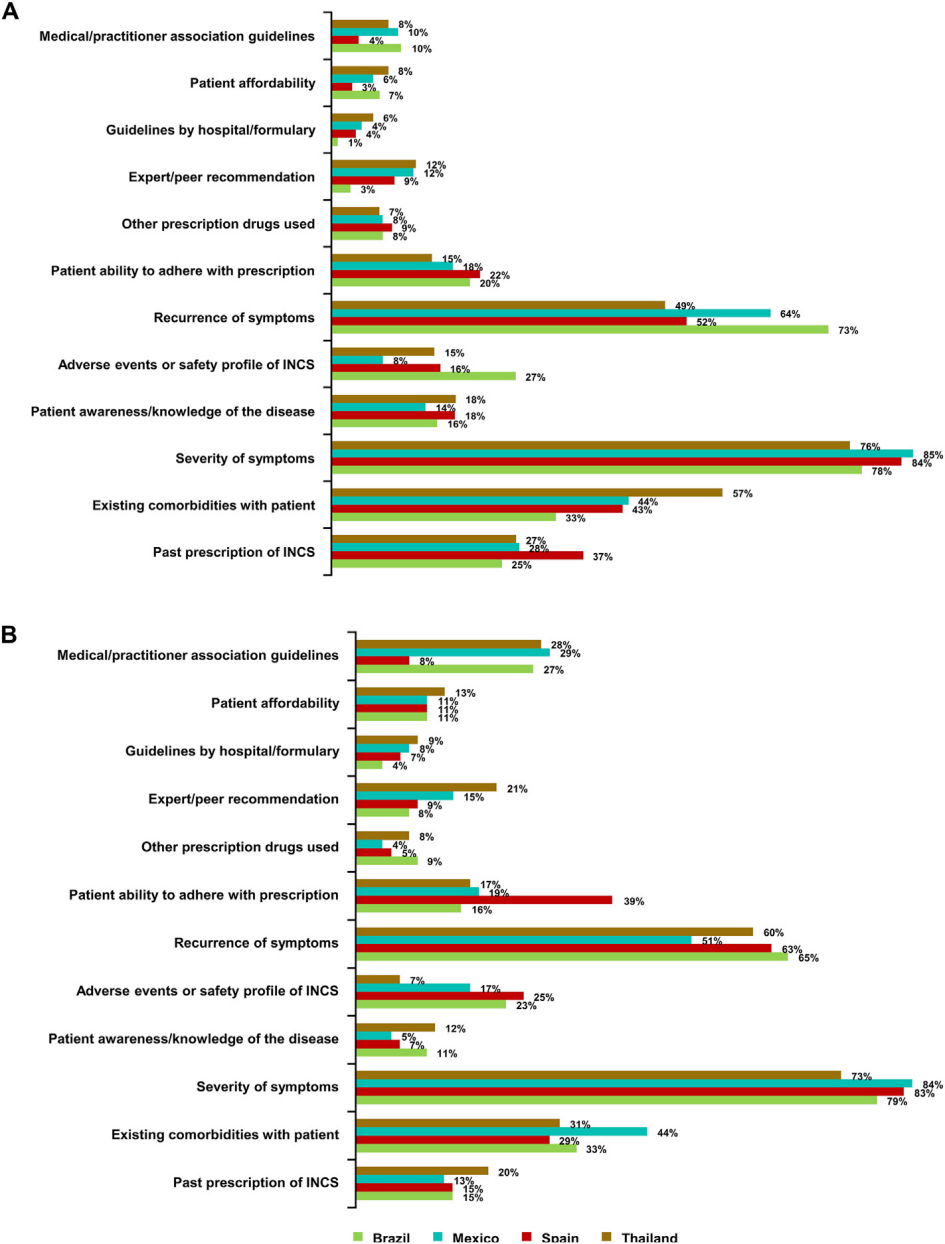
Factors influencing duration of INCS recommendation, according to GP-PRFs (A) and GP survey (B). GP, general practitioner; INCS, intranasal corticosteroid; PRF, patient record form

A total of 900 PRFs were collected from GPs (N = 225 for each country). The mean GP-PRF reported recommended INCS duration was 5.4–8.4 weeks across countries (Fig. 2). Severity of symptoms was the factor most frequently reported to influence the recommended INCS treatment duration in all 4 countries (76–85%) followed by symptom recurrence (49–73%) and existing comorbidities (33–57%) (Fig. 3A); symptoms and comorbidities are summarized in Supplementary Table 1.

**Fig. 4:**
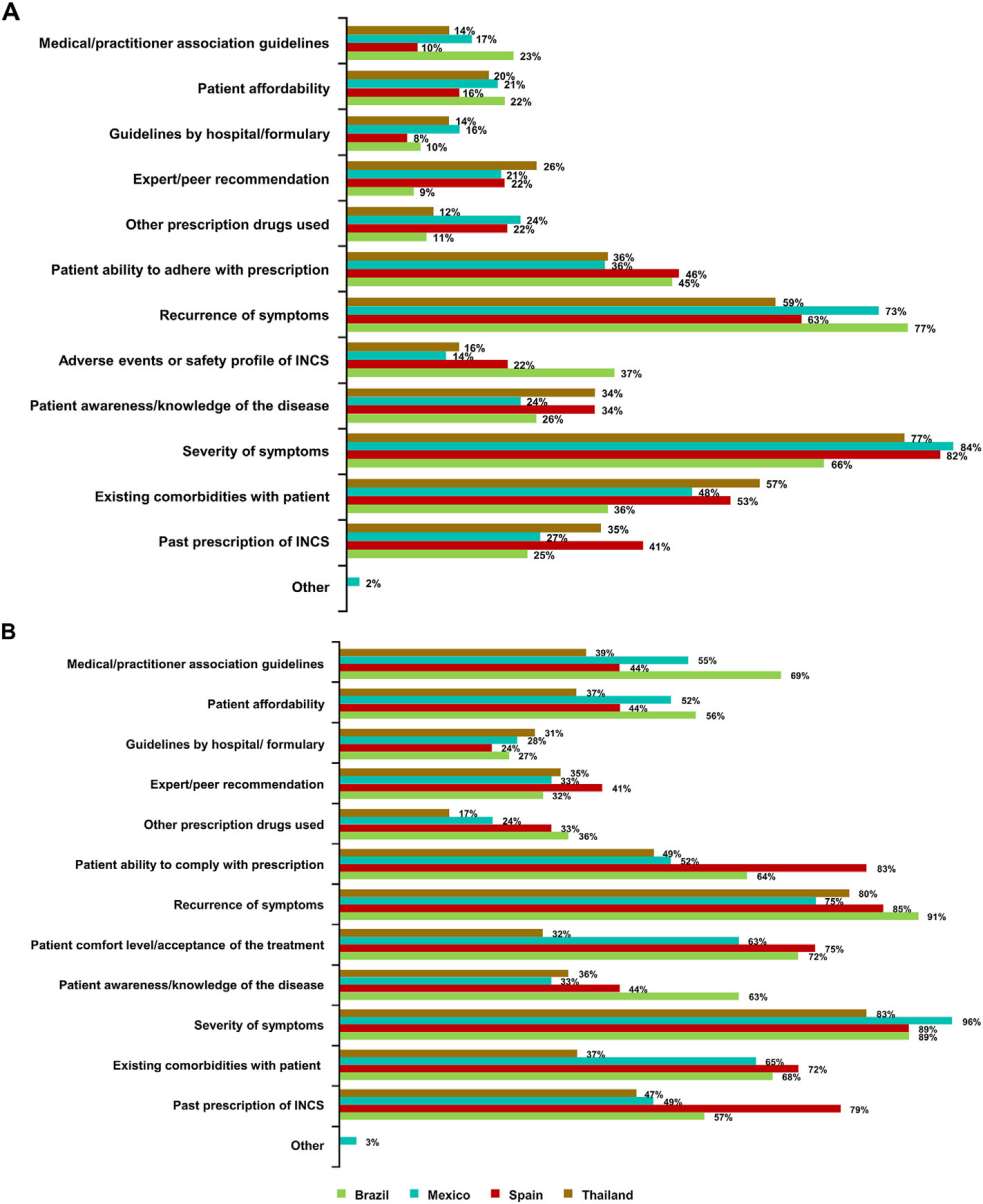
Reasons for recommending INCS, according to GP-PRFs (A) and GP survey (B). GP, general practitioner; INCS, intranasal corticosteroid; PRF, patient record form

The most frequently cited reasons for recommending INCS treatment across all countries included severity of symptoms (66–84%), symptom recurrence (59–77%), existing comorbidities (36–57%), and the ability of patients to adhere to the prescription (36–46%) (Fig. 4A).

**Fig. 5:**
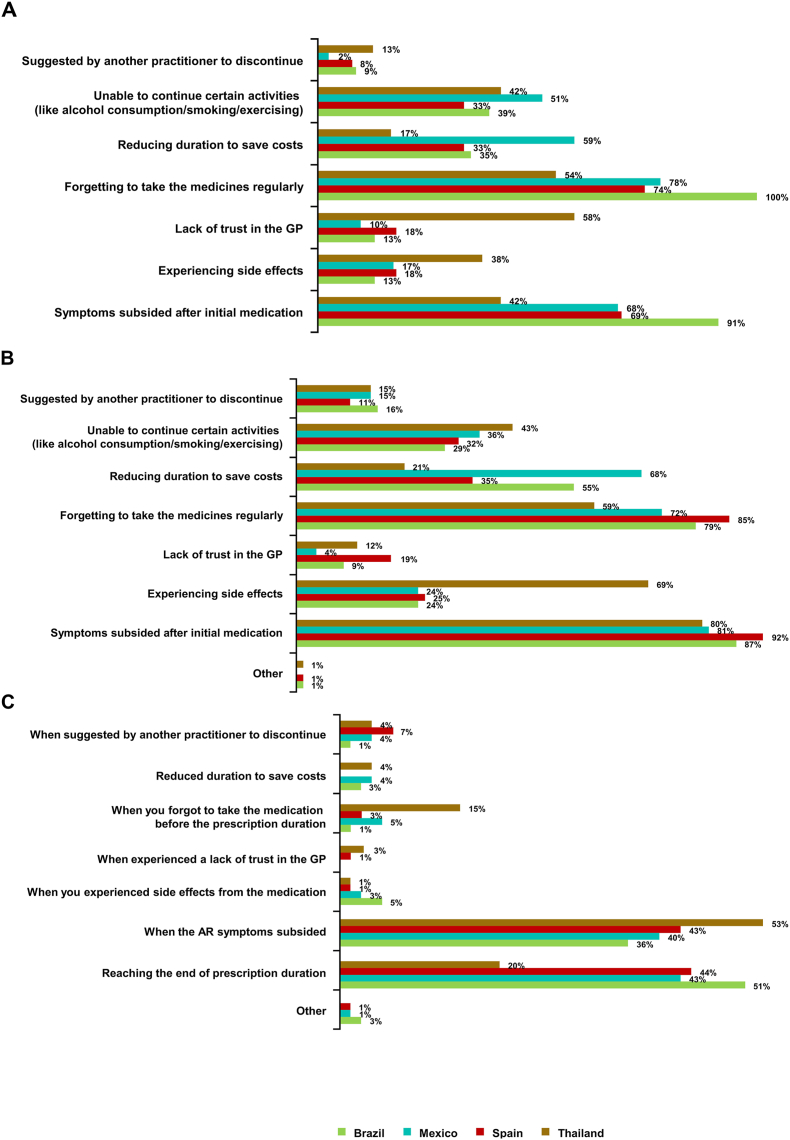
Factors influencing reasons for patient non-adherence to INCS, according to GP-PRFs (A) and GP survey (B); reasons for non-adherence to or discontinuation of INCS, according to patient survey (C). AR, allergic rhinitis; GP, general practitioner; INCS, intranasal corticosteroid; PRF, patient record form. Countries with responses of 0% are not shown

The factors most frequently reported by GP-PRFs as contributing to patient non-adherence across all countries (Fig. 5A) included forgetting to take medication regularly (54–100%), subsiding symptoms after initial medication (42–91%) and being unable to continue certain activities (such as alcohol consumption, smoking and/or exercise; 33–51%).

### GP survey findings

A total of 300 surveys were completed by GPs (N = 75 for each country). Symptom severity was the factor most frequently reported to influence INCS prescription durations in all participating countries (73–84%; Fig. 3B), similar to findings from the GP-PRF data (Fig. 3A); symptoms are summarized in Supplementary Table 1. GPs predominantly classified AR severity using their clinical judgment (72% Brazil, 53% Mexico, 71% Spain, 69% Thailand), ARIA guidelines (53% Brazil, 73% Mexico, 49% Spain, 37% Thailand), or the visual analogue scale (32% Brazil, 31% Mexico, 39% Spain, 11% Thailand).

Frequently reported reasons for recommending INCSs to patients with AR included severity of symptoms (83–96%), symptom recurrence (75–91%), existing comorbidities (37–72%), the ability of patients to adhere to the prescription (49–83%) and patient comfort level or acceptance of the treatment (32–75%) (Fig. 4B).

Subsiding symptoms after initial medication was the leading factor reported to influence patient non-adherence in all participating countries (80–92%) (Fig. 5B), which mirrors the GP-PRF findings for Brazil, Mexico, and Spain (Fig. 5A).

GPs in all countries reported choosing to continue with the same treatment at a decreased dose (49–55%) or continue with the same treatment and dosing (13–43%) when patients respond positively to their prescribed INCS. When patients fail to respond to their prescribed INCS, GPs reported proceeding by checking patient adherence (9–51%) or referring them to a specialist (0–51%).

### Patient survey

The patient survey recruited 300 patients. The most common reported symptoms experienced at presentation for patients across all countries were sneezing (76%), nasal stuffiness/congestion (76%), itchy nose (73%) and runny nose (71%) (Supplementary Table 1).

The mean patient-reported recommended INCS durations ranged from 4.0 weeks in Spain to 6.4 weeks in Brazil, and the mean patient-reported INCS use durations ranged from 3.6 weeks in Spain to 6.4 weeks in Thailand (Fig. 2).

The most frequently reported reasons for INCS non-adherence or discontinuation were subsiding symptoms (36–53%) and reaching the end of the prescription duration (20–51%) (Fig. 5C). Approximately 50% of patients who stopped INCSs before the end of their prescribed treatment duration experienced recurrence of symptoms. Many of these patients reported that they visited a different physician or the pharmacy for help with recurring symptoms; most patients in Thailand (83%) and some in Brazil (19%) also chose to self-medicate with a previously prescribed INCS therapy.

## Discussion

In this study, the recommended durations of INCS treatment ranged from 5.4 to 8.4 weeks, as reported by GPs, and 4.0–6.4 weeks, as reported by patients. The patient-reported durations of INCS use were shorter, ranging from 3.6 to 6.4 weeks.

Symptom severity was the factor most frequently reported to influence the GP-recommended duration of INCS treatment, ranging from 76 to 85% of GP-PRF responses and 73–84% of GP survey responses across participating countries. Severity of symptoms was also among the most frequent reasons GPs reported for recommending INCS treatment for AR, in addition to symptom recurrence, existing comorbidities, and the ability of patients to adhere to the prescription. Symptom severity is a key factor to consider according to ARIA treatment recommendations;^2^ however, the findings of an international survey study highlight key differences in patient and physician perspectives on the severity of symptoms in AR.^11^ The same study also reported significant (p < 0.001) variation across countries in the length of use for prescribed AR-INCSs, indicating that approximately half of patients in Brazil, Japan, and Spain only used INCSs during the allergy season, and approximately half of patients in Saudi Arabia used INCSs most of the time.^11^ Despite differences in seasonality, across all countries in our study three quarters of patients consistently reported having AR symptoms over multiple months, while the remaining quarter reported symptoms for a few days or weeks during each episode of symptoms.

The shortest recommended durations of INCS treatment (reported by both GPs and patients), as well as the shortest patient-reported durations of INCS use, were in Spain. This may reflect practices in the Spanish public healthcare system, where physicians have guidelines on duration and type of treatment prescribed and exercise caution in prescribing. Physicians in Spain generally report good knowledge of, and adherence to, ARIA guidelines (93% and 90%, respectively).^12^ In our survey, however, most GP respondents in Spain, Brazil, and Thailand (69–72%) reported using clinical judgment to assess disease severity rather than specifically adhering to clinical guidelines.

Many patients in Brazil, Mexico, and Spain recalled a shorter prescription duration than reported by physicians and the actual duration of use was even lower, indicating a tendency to not adhere to the prescribed treatment duration. Patients in Thailand reported shorter prescription durations than physicians but also reported longer periods of use, closer to the timeframes prescribed by local GPs. These patients likely prolonged their INCS use due to the AR symptoms they experienced. When patients fail to respond to INCSs, approximately half of GPs in Spain (but a much lower percentage of GPs in other countries) reported that they check patient adherence, while in Thailand the same proportion of GPs reported that they refer to a specialist.

Across all countries, subsiding symptoms, followed by forgetting to take medication, were the leading GP-reported factors influencing patient non-adherence to treatment. These factors are linked, as symptom subsidence may lead to patients forgetting to take medication. In contrast, the leading patient-reported factor leading to non-adherence, with the exception of patients in Thailand, was reaching the end of the prescription duration (even though actual reported use did not always reflect the prescribed duration). Previously reported reasons for non-adherence include forgetting to take medications, perceived lack of benefit, fear of side effects, and inconvenience.^13^, ^14^, ^15^, ^16^ Greater consensus on treatment duration between physicians and patients is needed because non-adherence to the prescribed treatment duration can lead to disease recurrence.

Approximately 50% of patients in our study who reported stopping INCSs before the prescribed duration experienced symptom recurrence, which included an increased frequency of allergic reactions and duration of disease which impacted their quality of life. Patients from all surveyed countries indicated that they visited the GP, a different physician, or the pharmacy to seek assistance for the recurrence of symptoms, while some patients also chose to self-medicate. This behaviour has been reported previously; patients may not take prescribed medications when symptoms are controlled and often co-medicate or consult their GP due to a perceived ineffectiveness of therapy when symptoms are uncontrolled.^2^^,^^17^ A more personalized approach to care may help to improve adherence among patients who would otherwise stop INCS therapy before the end of the prescribed treatment duration.

Strengths of this study include its multi-country design, gathering both GP- and patient-reported perspectives via surveys with corresponding PRF data from the same GPs, which identified drivers of INCS prescription. The consistency of findings across countries despite differences in seasonality during data collection and variability between medical systems is notable. For example, substantial differences were reported in the GP caseload per month (ranging from approximately 250 patients in Thailand to 650 patients in Spain) and in the proportions of patients diagnosed with AR (ranging from 15% in Thailand to 29% in Mexico). It is also interesting that both patients and GPs had confidence in INCS treatment and felt comfortable with its use. This is supported by a meta-analysis of 60 studies concluding that the use of INCS therapy was safe to use in adult populations.^18^ Another patient survey study indicated that although only 27% of patients with AR considered INCS use to be safe, most (71.5%) remained adherent to treatment despite these concerns.^19^

This study also has limitations. For example, survey results may have been subject to variation in AR-related factors, such as severity, prevalence, and the average GP caseload per country. Recall bias is also possible; as patients were asked to recall information regarding their previous prescription within the last three months of the questionnaire, it is possible that the patients may not exactly recall their actual duration of INCS use and reasons for non-adherence or discontinuation. However, GP-completed PRFs were reported as exactly prescribed to the patient, so these data were not subject to the same potential of recollection bias. Results of the patient survey from Thailand are largely from female patients (81% of respondents); views of male patients from Thailand may be under-represented. The patients surveyed were also not linked to the GPs surveyed and the patient survey data were likely more representative of AR populations in the investigated countries than the GP-PRF data. Finally, the study was not powered to statistically compare the differences between countries; hence, comparisons have not been drawn, and the small sample may not entirely reflect the populations of GPs and patients with AR present in clinical practice in each country. Noticeable trends to explore in future studies include differences in patient-reported factors impacting non-adherence in each country, such as mistrust in local physicians and reaching the end of prescription duration.

## Conclusion

This study gathered real-world GP and patient perspectives on the duration of INCS treatment for AR and obstacles to adherence from four countries with high AR prevalence. The actual duration of INCS treatment may be shorter than the prescribed duration due to non-adherence in some patients. Non-adherence may be influenced by factors including subsiding symptoms and forgetting to take medication. Many patients reported symptom recurrence despite INCS treatment, which could be linked to the potential for persistent minimal inflammation with a shortened therapy duration. A combination of techniques may provide a more personalized approach to improving adherence among patients using INCS therapy. Strategies to improve adherence could include patient counselling to promote autonomy and facilitate adherence to INCSs, and guidance for HCPs to understand modifiable patient barriers and patient preferences when taking INCSs. The investigation into the use of technological approaches to provide timely patient communications and interventions may also prevent early cessation of prescribed medications. Our study confirms data from previous studies with an App in patients with AR who tend to stop using their medication once symptoms are no longer present.^8^ In respiratory allergic disorders this same conduct was seen in asthma therapy, particularly among patients with less frequent symptoms. In asthma, global guidelines have finally adjusted to this patient conduct, leaving the therapy of milder cases on-demand (PRN), but stressing the importance to have an inhaled corticosteroid added to this PRN medication.^20^ A similar strategy could be used for AR, with INCS PRN use to avoid long-term self-medication with monotherapy of local or systemic vasoconstrictors, this may be valuable to investigate in confirmatory trials. Our findings also provide insight into the differences between patient and physician-reported outcomes and highlight the need for understanding between these groups to facilitate better treatment outcomes for patients with AR.

## Abbreviations

AR, allergic rhinitis; ARIA, Allergic Rhinitis and its Impact on Asthma; EUFOREA, European Forum for Research and Education in Allergy and Airway Diseases; GP, general practitioner; INCS, intranasal corticosteroid; PRF, patient record form; PRN, pro re nata (as needed); QC, quality check.

## Data availability statement

GSK makes available anonymized individual participant data and associated documents from interventional clinical studies which evaluate medicines, upon approval of proposals submitted to https://www.gsk-studyregister.com/en/. To access data for other types of GSK sponsored research, for study documents without patient-level data and for clinical studies not listed, please submit an enquiry via the website.

## Ethics statement

This study complied with all applicable laws regarding participant privacy and all participants provided informed consent. Following consideration of the study documents (protocol, survey, and consent form), ethical approval exemption was granted according to FDA 21 CFR 56.104 and 5CFR46.104(d)(2): Tests, Surveys, Interviews. The study was also reviewed by the Institutional Review Board and by the Human Research Ethics Committee at Khon Kaen Hospital in Thailand.

## Financial support

This study, including study design, data collection, analysis, and interpretation, and medical writing and submission support for the manuscript, was funded by GSK (study 217857). Medical writing support was provided by Jenni Lawton, PhD, of Ashfield MedComms (Macclesfield, UK), an Inizio company.

Trademarks are owned by or licensed to their respective owners (IQVIA network [IMS Health Incorporated], PASS [NCSS]).

## Agreement to publish the work

All authors agree to the publication of this work in the *World Allergy Organization Journal.*

## Statement of contribution to the work

All authors had access to the study data, take responsibility for the accuracy of the analysis, contributed to data interpretation, reviewed, and contributed to the content of the manuscript, and had authority in the decision to submit the manuscript.

## Editorial policy confirmation and agreement

All authors confirm they agree to World Allergy Organization's editorial policies.

## Confirmation of unpublished work

The authors confirm the following: their manuscript is original and has not been published in full before; is not currently being considered for publication elsewhere; and has not been posted to a preprint server. Some of the material discussed in this manuscript was previously presented at the World Allergy Congress 2023 (Bangkok, Thailand, 1–3 December 2023).

## Availability of data and materials

The authors confirm that the data supporting the findings of this study are available within the article and supplementary information.

## Declaration of competing interest

**DESL-L** reports personal fees from ALK, AstraZeneca, Bayer, Carnot, Chiesi Farmaceutici, Grin Therapeutics, Grünenthal, GSK, Menarini Group, Merck Sharp & Dohme (MSD), Novartis, Pfizer, Sanofi, Siegfried, Viatris; grants from Abbvie, AstraZeneca, Bayer, Circassia Group, GSK, Lilly, Sanofi, Novartis, Pfizer.

**PD** received grants for advisory boards and education activities from Chiesi, GSK, Guidotti and Sanofi in the last 2 years.

**RCDF** has no conflicts of interest to disclose.

**RGP** reports personal fees from AstraZeneca, Diater, GSK, Inmunotek, Leo Pharma and Sanofi outside the submitted work.

**MV** is an employee of and holds shares in GSK.
