# Supplementary material for: General practitioner and patient perspectives on intranasal corticosteroids for allergic rhinitis: Treatment duration and obstacles to adherence, findings from a recent survey
Source: World Allergy Organ J. 2024 Jun 25;17(7):100925. doi: 10.1016/j.waojou.2024.100925 (PMC11259959; doi:10.1016/j.waojou.2024.100925)
Supplement: Multimedia component 1 [file mmc1.pdf]

General practitioner and patient perspectives on intranasal corticosteroids for allergic rhinitis: treatment duration and obstacles to adherence, findings from a recent survey

SUPPLEMENTAL INFORMATION

1. The GP survey, completed by the general practitioners (GPs): Supplemental Appendix A – GP Survey
2. Patient record forms (PRFs), completed by the GPs: Supplemental Appendix B – GP patient Record Form
3. The patient survey, completed by individual patients with allergic rhinitis (AR): Supplemental Appendix C – Patient Survey
4. Supplementary Table 1. Summary of symptoms and comorbidities

1. Supplemental Appendix A – GP Survey

MAIN QUESTIONNAIRE

Section A – Patient Caseload

Q1

ASK ALL

PROGRAMMER:

- **NUMERIC RANGE [5-1000]**

In a typical month how many unique patients do you see **regardless of their condition**? **OPEN NUMERIC**

	**Number of patients**
**Total patient caseload in a typical month**	________

Q2

ASK ALL

PROGRAMMER:

- **NUMERIC RANGE [5-999]**

Of the **[INSERT Q1 Total]** patients you see in a typical month, how many are diagnosed with **any type of Allergic Rhinitis** (Intermittent/Persistent)? **OPEN NUMERIC**

		**Number of patients**
	**Total number of Allergic Rhinitis patients in a typical month**	________
		**Should be less than or equal to Q1 Total**
Q3	**ASK ALL**<br><br>**PROGRAMMER:**<br><br>• **NUMERIC RANGE [5-999]**<br><br>Thinking across all the patients you see in your practice, what is the total number of Allergic Rhinitis patients that are being **currently managed** by you? **OPEN NUMERIC**	

	**Number of patients**
**Total number of Allergic Rhinitis patients managed by GP**	________

Q4	**ASK ALL**<br><br>**PROGRAMMER:**<br><br>• **NUMERIC RANGE [5-999]**<br><br>Among the Allergic Rhinitis patients that you are managing, how many have you been managing for more than 1 year? **OPEN NUMERIC**

		**Number of patients**	
	**Total number of Allergic Rhinitis patients managed by GP for more than a year**	________	
		**Should be less than or equal to Q3 Total**	

Q5

ASK ALL

PROGRAMMER:

- **NUMERIC RANGE [5-999]**

Among the Allergic Rhinitis patients that you are managing for more than a year, how many have been initiated on INCS (Intra nasal corticosteroids) more than a year ago? **OPEN NUMERIC**

	**Number of patients**
**Total number of Allergic Rhinitis patients managed by GP for more than a year and initiated on INCS more than a year ago**	________
	**Should be less than or equal to Q4 Total**

Q5a

ASK ALL

- **MULTI SELECT**

	• RANDOMIZE OPTIONS<br><br>Can you please tell me that which of the following statements best describes your approach to classify severity of Allergic Rhinitis? **OPEN NUMERIC**

I don’t classify disease severity	1
I use VAS scale to classify disease severity	2
I use ARIA guidelines to classify disease severity	3
I use my Clinical judgement to classify disease severity	4
I use other methods such as _______(please specify)	5

Q6	**ASK ALL**<br><br>**Programmer:**<br><br>• **PERCENTAGE RANGE [0-100]**<br>• **SUM OF VALUES ACROSS ALL CATGEORIES SHOULD BE 100**<br>• **AUTOFILL 0**<br><br>Thinking of following groups of Allergic Rhinitis patients, in what proportion would you classify them under the following levels of severity?<br><br>Please use the below definitions to classify severity of Allergic Rhinitis:<br><br>• **Mild** – Patient are not exhibiting following symptoms: sleep disturbance, impairment of daily activities like leisure/sports/work, symptoms present but are not troublesome

	• **Moderate to Severe** – Patient are exhibiting following symptoms: sleep disturbance, impairment of daily activities/work, troublesome symptoms

Code	Severity Category	Q6a Allergic Rhinitis patients that were initiated on INCS (Intra nasal corticosteroids) more than a year ago **[Percentage]**	Q6b All Allergic Rhinitis patients that are being managed by you **[Percentage]**
**1**	Mild	____%	____%
**2**	Moderate to severe	____%	____%
	**TOTAL**	**AUTOSUM = 100%**	**AUTOSUM = 100%**

Q7

ASK ALL

Programmer:

- **PERCENTAGE RANGE [0-100]**
- **ADD DEFINITION FOR EACH CATEGORY**
- **DO NOT MOVE FORWARD UNTIL VALUES ARE FILLED FOR ALL PATIENT TYPES**

Thinking of following groups of Allergic Rhinitis patients, in what proportion would you classify them under the following types of Allergic Rhinitis?

	Please consider the following definitions to classify types of Allergic Rhinitis:<br><br>• **Intermittent/Seasonal:** symptoms are present less than or equal to 4 days a week or for less than or equal to 4 consecutive weeks<br>• **Persistent/Perennial:** symptoms are present more than 4 days a week or for more than 4 consecutive weeks

**Code**	**Type or Allergic Rhinitis**	Q7a Allergic Rhinitis patients that were initiated on INCS (Intra nasal corticosteroids) more than a year ago **[Percentage]**	Q7b All Allergic Rhinitis patients that are being managed by you **[Percentage]**
**1**	Persistent/Perennial Allergic Rhinitis	____%	____%
**2**	Intermittent/Seasonal Allergic Rhinitis	____%	____%
	**TOTAL**	**AUTOSUM = 100%**	**AUTOSUM = 100%**

Q8	**ASK ALL**<br><br>**Programmer:**<br><br>• **MULTI SELECT**

	Thinking of your Allergic Rhinitis patients who were initiated on INCS (Intra nasal corticosteroids) over a year ago, which of the following comorbidities do they suffer from?
Q9	**ASK ALL**

ASK ALL

Programmer:

- **PERCENTAGE RANGE [0-100]**
- **ONLY ASK FOR CATEGORIES SELECTED IN Q8**
- **DO NOT MOVE FORWARD UNTIL VALUES ARE FILLED FOR ALL PATIENT TYPES**

Thinking of your Allergic Rhinitis patients who were initiated on INCS (Intra nasal corticosteroids) over a year ago, what proportion of these patients have the following comorbidities?

Current Comorbidities of the patient	Q8	Q9
**Nasal Polyposis**	1	__%
**Asthma**	2	__%
**Atopic Dermatitis**	3	__%
**Sinusitis**	4	__%
**Otitis Media**	5	__%
**Conjunctivitis**	6	__%
**Others, please specify** ________________		

Section B – Prescription Behaviour and preferences

Please respond to all the questions in this section while thinking of your Allergic Rhinitis patients under management for at least 1 year and initiated on INCS (Intra nasal corticosteroids) at least 1 year ago

Q10

ASK ALL

PROGRAMMER:

- **MULTI SELECT**
- **RANDOMIZE OPTIONS**

Please select all the factors that you take into consideration when prescribing INCS (Intra nasal corticosteroids) treatment to your Allergic Rhinitis patients

Reasons for prescribing INCS treatment	Code
Past prescription of INCS (Intra nasal corticosteroids)	**1**
Existing comorbidities with patient	**2**
Severity of symptoms	**3**
Patient awareness/knowledge of the disease	**4**
Patient comfort level/acceptance of the treatment	**5**
Recurrence of symptoms	**6**
Patient ability to comply with prescription	**7**

Other prescription drugs used	**8**
Expert/Peer recommendation	**9**
Guidelines by hospital/formulary	**10**
Patient affordability	**11**
Medical/Physician association guidelines	**12**
Other (please specify_______)	**99**

Q11

ASK ALL

PROGRAMMER:

- **SINGLE SELECT**
- **RANDOMIZE OPTIONS**

After Allergic Rhinitis diagnosis is confirmed, which of the below mentioned points best describe the stage at which INCS (Intra nasal corticosteroids) treatment is most often prescribed?

**Stage at which INCS (Intra nasal corticosteroids) treatment is most often prescribed**	**Code**
Immediately after diagnosis	**1**
If severity of symptoms increases	**2**
If symptoms are persistent	**3**
If response is poor to antihistamines	**4**

Q12

ASK

PROGRAMMER:

- **OPEN NUMERIC [RANGE 1 – 9999]**
- **DO NOT MOVE FORWARD UNTIL VALUES ARE FILLED FOR ALL PATIENT TYPES**
- **IF Q6a CODE 1 = 0 , SHOW ONLY ROW WITH CODE 2 – Moderate to Severe**

Thinking of your Allergic Rhinitis patients who were initiated on INCS (Intra nasal corticosteroids) over a year ago, can you please mention the average duration for which INCS was **prescribed by you for latest AR episode at least 3 months ago?** Please respond for each of the Allergic Rhinitis severity levels below:

Mild – Patient are not exhibiting following symptoms: sleep disturbance, impairment of daily activities like leisure/sports/work, symptoms are not troublesome

Moderate to Severe – Patient are exhibiting following symptoms: sleep disturbance, impairment of daily activities/work, troublesome symptoms

Code	Severity Category	Average duration of INCS (Intra nasal corticosteroids) treatment for latest AR episode at least 3 months ago
**1**	Mild	____Weeks
**2**	Moderate to Severe	____ Weeks

20

Q13 **ASK ALL**

PROGRAMMER:

- **RANKING UPTO TOP 3**
- **RANDOMIZE OPTIONS**

Please rank the factors that you usually take into consideration when **deciding on the duration of INCS** (Intra nasal corticosteroids) treatment to Allergic Rhinitis patients. Among the factors, please **rank up to the 5 most important factors** starting with ‘1’ as ‘most important’, ‘2’ as ‘2nd most important’, and ‘3’ as ‘3rd most important

**Factors influencing duration of INCS (Intra nasal corticosteroids) treatment**	**Code**
Past prescription of INCS (Intra nasal corticosteroids)	**1**
Existing comorbidities with patient	**2**
Severity of symptoms	**3**
Patient awareness/knowledge of the disease	**4**
Adverse events or safety profile of INCS	**5**
Recurrence of symptoms	**6**
Patient ability to comply with prescription	**7**
Other prescription drugs used	**8**

	Expert/Peer recommendation	**9**
	Guidelines by hospital/formulary	**10**
	Patient affordability	**11**
	Medical/Physician association guidelines	**12**
	INSERT OPTION TYPED IN CODE 99 Q10	**13**
Q14	**ASK**<br><br>**PROGRAMMER:**<br><br>• **OPEN NUMERIC [RANGE 1 – 9999]**<br>• **DO NOT MOVE FORWARD UNTIL VALUES ARE FILLED FOR ALL PATIENT TYPES**<br>• **IF Q6a CODE 1 = 0 , SHOW ONLY ROW WITH CODE 2 – Moderate to Severe**<br><br>**As per the policy & guideline of your current practise setting, what is the recommended prescription duration for prescribing INCS treatment to patients diagnosed with Allergic Rhinitis for the patients with the following levels of severity**<br><br>**Mild** – Patient are not exhibiting following symptoms: sleep disturbance, impairment of daily activities like leisure/sports/work, symptoms are not troublesome<br><br>**Moderate to Severe** – Patient are exhibiting following symptoms: sleep disturbance, impairment of daily activities/work, troublesome symptoms	

Code	Severity Category	Recommended duration of INCS (Intra nasal corticosteroids) treatment
**1**	Mild	____Weeks
**2**	Moderate to Severe	____ Weeks

Q15

ASK ALL

PROGRAMMER:

- **SINGLE SELECT**
- **RANDOMIZE OPTIONS**

What is your most likely course of action after an Allergic Rhinitis **patient responds positively to INCS** (Intra nasal corticosteroids) treatment?

Response	Code
Continue with same treatment and **same dosing**	**1**
Continue with same treatment but with an **increased dose**	**2**
Continue this treatment and would **add another treatment as an add on therapy**	**3**
**Discontinue** this treatment and would **switch to** another treatment	**4**
**Discontinue** all treatments	**5**

Q16

ASK ALL

PROGRAMMER:

- **MULTI SELECT**
- **RANDOMIZE OPTIONS**

What is your most likely course of action after an Allergic Rhinitis patient **fails to respond to the INCS** (Intra nasal corticosteroids) treatment?

**Response**	**Code**
Continue with same treatment and **same dosing for some more time**	**1**
Continue with same treatment but with an **increased dose**	**2**
Continue this treatment and would **add another treatment as an add on therapy**	**3**
**Discontinue** this treatment and would **switch to** another treatment	**4**
**Refer** to a specialist	5
**Check patient compliance** with therapy prescribed	6
**Re-educate patient** on how to use the Nasal spray device	7

Q17a	**ASK ALL**<br><br>**PROGRAMMER:**<br><br>• **MULTI SELECT**<br>• **RANDOMIZE OPTIONS**<br><br>According to you, what are the **reasons for which INCS** (Intra nasal corticosteroids) treatment fails in Allergic Rhinitis patients?

Reasons for INCS (Intra nasal corticosteroids) treatment failure	Code
Low dosage	1
Low duration prescribed	2
Lack of patient compliance to treatment	3
Others (Please Specify______)	

Q17	**ASK ALL**<br><br>**PROGRAMMER:**<br><br>• **RANKING UPTO TOP 3**<br>• **RANDOMIZE OPTIONS**<br><br>According to you, what are the top 3 reasons for Allergic Rhinitis patients prescribed on INCS (Intra nasal corticosteroids) to **not adhere to the prescribed**

	**treatment duration**?<br><br>Among the factors, please **rank up to the 3 most important factors** starting with '1' as 'most important', '2' as '2nd most important', and '3' as '3rd most important

**Factors responsible for lack of patient compliance**	**Code**
Symptoms subsided after initial medication	**1**
Experiencing side effects	**2**
Lack of trust in the General Physician	**3**
Forgetting to take the medicines regularly	**4**
Reducing duration to save costs	**5**
Unable to continue certain activities<br><br>(like alcohol consumption/smoking/exercising)	**6**
Suggested by another Physician to discontinue	**7**
Other (Please Specify_____)	

Q18	**ASK ALL**<br><br>**PROGRAMMER:**<br><br>• **MULTI SELECT**<br>• **RANDOMIZE OPTIONS**<br><br>According to you, what are some of the most common **outcomes caused by lack of adherence** by Allergic Rhinitis patients for INCS (Intra nasal corticosteroids) prescription duration?

	Code
Increase in disease severity	**1**
Increase in frequency of allergic reactions	**2**
Increased disease duration	**3**
Reduced in patient quality of life	**4**
Investigations prescribed for further diagnosis	**5**
Other (Please Specify_____)	

Q19

ASK ALL

PROGRAMMER:

- **MULTI SELECT**
- **RANDOMIZE OPTIONS**

Please indicate what factors do you usually take into consideration **when discontinuing INCS** (Intra nasal corticosteroids) among Allergic Rhinitis patients?

Among the factors, please rank **up to the top 3 most important factors** starting with '1' as 'most important', '2' as '2nd most important', and '3' as '3rd most important.

Code	Factors for discontinuing INCS (Intra nasal corticosteroids) treatment	Rank

**1**	Improvement in patient condition (symptoms in control/no recurrence)	
**2**	INCS treatment side-effects	
**3**	Patient experiencing adverse event of INCS (Intra nasal corticosteroids)	
**4**	Inadequate impact on disease progression	
**5**	Poor patient compliance	

Q20

ASK ALL

PROGRAMMER:

- **MULTI SELECT**
- **RANDOMIZE OPTIONS**

Please indicate what factors do you usually take into consideration **when referring a patient to a specialist?**

**Code**	**Factors for referring a patient**	**Code**
1	Due to comorbidities	1
2	Increase in disease severity	2
3	Patient’s request for referral	3
4	To explore other treatment options	4

	5	Suspicion of other conditions	5	
	6	Others, please specify (______)		

23

2. Supplemental Appendix B – GP Patient Record Form

ALLERGIC RHINITIS PATIENT CASE COLLECTION

We would now like to discuss with you some real patient cases.

We would like you to think of the MOST RECENT 3 **Allergic Rhinitis** adult patients (≥18 years old) seen by you in the last 3 months and who have been under your management for at least one year and were first prescribed **Intra nasal corticosteroid (INCS)** at least 1 year ago. It is important for us that you think back to precisely identify the last 3 Allergic Rhinitis patients you managed, regardless of whether this was a new case or a patient who was referred to you, and not one which you may consider to be more interesting. Once you have identified the 3 patients who meet these criteria, please move to the next section, and answer the following set of questions. Each set of question will be repeated 3 times, for each set please respond to the questions for one of the 3 shortlisted patients.

<table>
<tr><td colspan="2">Patient Record Form: Patient 1</td></tr>
<tr><td>P1. What is the patient’s sex?

SINGLE SELECT

1. Female
2. Male</td><td>P2. What is the patient’s current age?

SINGLE SELECT

1. From 18 to 34 years old
2. From 35 to 49 years old
3. From 50 to 64 years old
4. ≥ 65 years old</td></tr>
<tr><td colspan="2">P3a. When was this patient first diagnosed with AR?</td></tr>
</table>

<table>
<tr><td colspan="2">SINGLE SELECT

1. From 1-1.5 years ago
2. From 1.5-3 years ago
3. From 3 to 4 years ago
4. More than 4 years ago</td></tr>
<tr><td>P3b. When was INCS first prescribed to this patient?

SINGLE SELECT

1. From 1-1.5 years ago
2. From 1.5-3 years ago
3. From 3 to 4 years ago
4. More than 4 years ago</td><td>P3c. When was the last follow up with this patient?

SINGLE SELECT

1. One Month ago
2. Two Months ago
3. Three Months ago
4. More than three months ago</td></tr>
<tr><td colspan="2">P4. Does the patient have any of the following comorbidities?

MULTI SELECT

1. Nasal Polyposis</td></tr>
</table>

2. Asthma
3. Atopic Dermatitis
4. Sinusitis
5. Otitis Media
6. Conjunctivitis
7. Others, please specify_______

P5. What is the level of severity of Allergic Rhinitis in this patient?

SINGLE SELECT

Please use the below definitions to classify severity of Allergic Rhinitis:

- **Mild** – Patient are not exhibiting following symptoms: sleep disturbance, impairment of daily activities like leisure/sports/work, symptoms present but are not troublesome
- **Moderate to Severe** – Patient are exhibiting following symptoms: sleep disturbance, impairment of daily activities/work, troublesome symptoms

1. Mild
2. Moderate to severe

P6. How would you classify this patient in terms of type of Allergic rhinitis?

SINGLE SELECT

Please consider the following definitions to classify types of Allergic Rhinitis:

• **Intermittent/Seasonal:** symptoms are present less than or equal to 4 days a week or for less than or equal to 4 consecutive weeks<br>• **Persistent/Perennial:** symptoms are present more than 4 days a week or for more than 4 consecutive weeks<br><br>1. Persistent/Perennial Allergic rhinitis<br>2. Intermittent/Seasonal Allergic rhinitis
P7. What symptoms did the patient experience at presentation? Please select all those apply<br><br>MULTI SELECT<br><br>1. Nasal stuffiness (congestion)<br>2. Sneezing<br>3. Runny Nose<br>4. Itchy Nose<br>5. Itching in throat & eyes<br>6. Headache<br>7. Sinus pain<br>8. Increased mucus generation in nose and throat<br>9. Fatigue<br>10. Malaise (general feeling of discomfort)<br>11. Sleep loss due to discomfort

12. **Others, please specify_____**
P8. What was the previous medication which the patient was using for Allergic Rhinitis prior to your INCS prescription?<br><br>MULTI SELECT<br><br>1. No previous medication<br>2. Antihistamines<br>3. Intra nasal corticosteroids (INCS)<br>4. Others, please specify_____
P9. Please select all the factors that you took into consideration when prescribing INCS (Intra nasal corticosteroids) treatment to this Allergic Rhinitis patient?<br><br>MULTI SELECT<br><br>1. Past prescription of INCS (Intra nasal corticosteroids)<br>2. Existing comorbidities with patient<br>3. Severity of symptoms<br>4. Patient awareness/knowledge of the disease<br>5. Adverse events or safety profile of INCS<br>6. Recurrence of symptoms<br>7. Patient ability to comply with prescription<br>8. Other prescription drugs used<br>9. Expert/Peer recommendation

10. Guidelines by hospital/formulary<br>11. Patient affordability<br>12. Medical/Physician association guidelines<br>13. Other, please specify_____
P10. Can you please mention the duration for which INCS was **prescribed by you for the latest prescription** to this patient? [open numeric]<br><br>• P10: Latest prescription - ____Weeks
P11. Please rank the factors that you usually take into consideration when deciding on the duration of INCS (Intra nasal corticosteroids) treatment to Allergic Rhinitis patients. Please rank top 3 most important factors starting with '1' as 'most important', '2' as '2nd most important', and '3' as '3rd most important<br><br>RANKING UPTO TOP 3<br><br>1. Past prescription of INCS (Intra nasal corticosteroids)<br>2. Existing comorbidities with patient<br>3. Severity of symptoms<br>4. Patient awareness/knowledge of the disease<br>5. Adverse events or safety profile of INCS<br>6. Recurrence of symptoms<br>7. Patient ability to comply with prescription<br>8. Other prescription drugs used

9. Expert/Peer recommendation
10. Guidelines by hospital/formulary
11. Patient affordability
12. Medical/Physician association guidelines
13. INSERT OPTION ENTERED IN P9 CODE 13

P12. What was the outcome of the latest INCS prescription you made to the patient?

SINGLE SELECT

1. Patient positively responded to the prescription
2. Patient failed to respond to the prescription

P13. Did the patient completely adhere to the prescription duration prescribed by you?

SINGLE SELECT

1. Yes
2. No

IF YES coded at P13

P13a. Did you stop the INCS treatment before the prescribed duration?

1. Yes
2. No

IF YES coded at P13a

P13b. Why did you stop INCS treatment before the prescribed duration?

1. Patient had side effects
2. Patient was unable to properly use INCS
3. Switched to a different type of medication
4. Others (Please Specify_____)

If NO coded in P13

P14. According to you, what are the reasons for this allergic rhinitis patient to not adhere to the prescribed treatment duration of INCS (Intra nasal corticosteroids)? Please rank top 3 most important factors starting with ‘1’ as ‘most important’, ‘2’ as ‘2nd most important’, and ‘3’ as ‘3rd most important

RANKING UPTO TOP 3

1. Symptoms subsided after initial medication
2. Experiencing side effects
3. Lack of trust in the General Physician
4. Forgetting to take the medicines regularly
5. Reducing duration to save costs
6. Unable to continue certain activities (like alcohol consumption/smoking/exercising)
7. Suggested by another physician to discontinue
8. I am not aware of the reasons (SINGLE SELECT)

If NO coded in P13

P15. What were the outcomes caused by lack of adherence to INCS (Intra nasal corticosteroids) prescription duration to this Allergic Rhinitis patient?

MULTI SELECT

1. Increase in disease severity
2. Increase in frequency of allergic reactions
3. Increased disease duration
4. Reduced in patient quality of life
5. Investigations prescribed for further diagnosis
6. Others, please specify____

Randomize options

P16. Which of the following statements describe the actions you take to improve the patient adherence to prescription duration which you prescribed?

Please rank top 3 most important factors starting with ‘1’ as ‘most important’, ‘2’ as ‘2nd most important’, and ‘3’ as ‘3rd most important’

MULTI SELECT

1. I don’t take any action to improve patient compliance
2. Provide individual counselling
3. Share material for self-reading
4. Recommend consultation in shorter intervals
5. Provide compliance charts
6. Modify prescription considering the financial burden
7. Others________

36

3. Supplemental Appendix C – Patient Survey

MAIN QUESTIONNAIRE

Section A – Patient Treatment

Q1	**ASK ALL**<br><br>**PROGRAMMER:**<br><br>• **MULTI SELECT**<br>• **RANDOMIZE OPTIONS**<br><br>Thinking of the most recent episode of Allergic Rhinitis at least 3 months ago, which of the following steps did you take to deal with the symptoms?

**Steps taken**	**Code**
Did not take any action	**1**
Started non-medicinal treatment (exercise/herbal products/home remedies)	**2**
Started medication from my own knowledge (self-medication)	**3**
Started medication recommended by a local pharmacist	**4**
Visiting a General Physician	**5**

		Visited a specialist (Allergist/ENT/Pulmonologist)	**6**	
		Others (Please Specify____)		

Q2

ASK ALL

PROGRAMMER:

- **MULTI SELECT**
- **RANDOMIZE OPTIONS**

Thinking of the most recent episode of Allergic Rhinitis at least 3 months ago, which symptoms were you experiencing when you decided to visit a general physician for the condition?

**Symptoms**	**Code**
Nasal stuffiness (congestion)	**1**
Sneezing	**2**
Runny Nose	**3**
Itchy Nose	**4**
Itching in throat & eyes	**5**
Headache	**6**
Sinus pain	**7**
Increased mucus generation in nose and throat	**8**

		Fatigue	**9**
		Malaise (general feeling of discomfort)	**10**
		Sleep loss due to discomfort	**11**
		Others (please specify____)	

Q3a **ASK ALL**

Programmer:

- **SINGLE SELECT**
- **INSERT LIST OF DRUGS SELECTED IN S7**

Please identify the most recent Intra Nasal Corticosteroid (INCS) drug prescribed to you at least 3 months ago from the list of INCS sprays you have used in the past?

**Event**	**Code**
Avamys	**1**
Rhinocart	**2**
Nasonex	**3**

Q3 **ASK ALL**

	**Programmer:**<br><br>• **DURATION IN WEEKS**<br>• **INSERT DRUG SELECTED IN Q3a**<br><br>Thinking of most recent prescription which you received at least 3 months ago for Allergic Rhinitis**,** can you please mention, for how long was **[INSERT DRUG SELECTED IN Q3a]** prescribed by the doctor?
Q4	**ASK ALL**

		**Duration of most recent prescription at least 3 months ago**	
1	**INSERT DRUGS SELECTED IN Q3a**	____weeks	

	**Programmer:**<br><br>• **SINGLE SELECT**<br>• **RANDOMIZE OPTIONS**<br><br>Thinking of your most recent prescription of **[INSERT DRUG SELECTED IN Q3a]** which you received at least 3 months ago, at what point did you stop taking **[INSERT DRUG SELECTED IN Q3a]**?

Event	Code
When the Allergic Rhinitis symptoms subsided	**1**
When the prescribed duration got over	**2**
When you experienced side effects from the medication	**3**
When you forgot to take the medication before the prescription duration	**4**
Suggested by another Physician to discontinue	**5**
Lack of trust in the General Physician	**6**
Reduced duration to save costs	**7**

	Others, please specify (________)	
Q5	**ASK ALL**<br><br>**Programmer:**<br><br>• **DURATION IN WEEKS**<br>• **INSERT DRUG SELECTED IN Q3a**<br><br>Thinking of your most recent prescription of **[INSERT DRUG SELECTED IN Q3a]** which you received at least 3 months ago, can you please mention how long you used **[INSERT DRUG SELECTED IN Q3a]** Please mention about the actual duration for which the drug was used.	
Q6	**ASK ONLY IF DURATION STATED IN Q5 IS LESS THAN DURATION IN Q3**	

	**INSERT DRUGS SELECTED IN Q3a**	**Duration of actual use of most recent prescription received at least 3 months ago**
1	**INSERT DRUGS SELECTED IN Q3a**	____weeks

	**Programmer:**<br><br>• **SINGLE SELECT**<br>• **INSERT DRUG SELECTED IN Q3a**<br><br>Did you experience recurrence of any symptoms of Allergic Rhinitis after stopping **[INSERT DRUG SELECTED IN Q3a]** before the prescribed duration of **[INSERT DURATION FROM Q3]**?<br><br><table><tr><th></th><th>Code</th></tr><tr><td>Yes</td><td>**1**</td></tr><tr><td>No</td><td>**2**</td></tr></table>
Q7	**ASK ONLY IF DURATION STATED IN Q5 IS LESS THAN DURATION IN Q3**<br><br>**Programmer:**<br><br>• **MULTI SELECT**<br>• **INSERT LIST OF DRUGS SELECTED IN Q3a**<br><br>You mentioned that you stopped using **[INSERT DRUG SELECTED IN Q3a]** after **[INSERT DURATION FROM Q5]**<br><br>After you stopped, which of the following did you experience related to your Allergic Rhinitis condition?<br><br><table><tr><th>Patient Experiences</th><th>Code</th></tr></table>

	Increase in disease severity	**1**
	Increase in frequency of allergic reactions	**2**
	Increased disease duration	**3**
	Reduction in patient quality of life	**4**
	Investigations prescribed for further diagnosis	**5**
	None of the above	**98**
Q8	**ASK ONLY IF DURATION STATED IN Q5 IS LESS THAN DURATION IN Q3** <br>**Programmer:** <br>• **MULTI SELECT** • **INSERT DRUG SELECTED IN Q3a** <br>You mentioned that you stopped using **[INSERT DRUG SELECTED IN Q3a]** after **[INSERT DURATION FROM Q5]**. Did you experience any of the following symptoms after your stopped?	

**Symptoms**	**Code**
No symptom experienced *(single code)*	**1**
Nasal stuffiness (congestion)	**2**
Sneezing	**3**

		Runny Nose	**4**
		Itchy Nose	**5**
		Itching in throat & eyes	**6**
		Headache	**7**
		Sinus pain	**8**
		Increased mucus generation in nose and throat	**9**
		Fatigue	**10**
		Malaise (general feeling of discomfort)	**11**
		Sleep loss due to discomfort	**12**

Q9

ASK ONLY IF RESPONSE IN Q6 = 'Code 1'

Programmer:

- **SINGLE SELECT**
- **RANDOMIZE OPTIONS**
- **ASK IF 01 is not coded in Q09**

What was your next step after experiencing recurrence of symptoms of Allergic Rhinitis?

Next Steps	Code

Visiting a General Physician	**1**
Visiting pharmacy for self-medication	**2**
Self-medicate with **[INSERT DRUGS SELECTED IN Q3]**	**3**
Take no action	**4**
Visit a different physician	**5**
Others________	**99**

Q10 **ASK ALL**

PROGRAMMER:

- **MULTI SELECT**
- **RANDOMIZE OPTIONS**

You mentioned that you are currently visiting General Physician for your Allergic Rhinitis condition, which of the following are the key reasons for you to regularly visit a general physician?

Reasons for visiting GP	Code

	For INCS (Intra nasal corticosteroids used in the form of nasal spray) prescription	**1**
	As part of regular visits	**2**
	Felt the symptoms to be unmanageable	**3**
	To ensure that the symptoms do not intensify further and remain manageable	**4**
	Others________	**99**

**Section B – Patient Profile**

Q11	**ASK IF S6b = CODE 3**<br><br>**Programmer:**<br><br>• **SINGLE SELECT**<br><br>You mentioned that you visited a specialist in last 1 year for your Allergic Rhinitis condition, which of the following was the most important factor for your visit to specialist?

**CONDITION**	**Code**

My General Physician referred me to a specialist	**1**
I requested my General Physician for a specialist consultation	**2**
I directly visited a specialist, without referral	**3**

Q12 **ASK ALL**

Programmer:

- **SINGLE SELECT**

Please indicate your working status

WORKING STATUS	Code
Full time employee	**1**
Part time employee	**2**
Self-employed	**3**
Retired	**4**
Unemployed	**5**

42

43

4. Supplementary Table 1. Summary of symptoms and comorbidities

Thinking of your AR patients who were initiated on INCS over a year ago, which of the following comorbidities do they suffer from? n (%)	GP survey responses			
	Thailand (N=75)	Brazil (N=75)	Spain (N=75)	Mexico (N=75)
Nasal polyposis	30 (40)	50 (67)	64 (85)	42 (56)
Asthma	67 (89)	70 (93)	73 (97)	66 (88)
Atopic dermatitis	62 (83)	56 (75)	65 (87)	55 (73)
Sinusitis	55 (73)	67 (89)	59 (79)	63 (84)
Otitis media	17 (23)	38 (51)	29 (39)	39 (52)
Conjunctivitis	35 (47)	39 (52)	61 (81)	50 (67)
Sleep apnea	25 (33)	44 (59)	37 (49)	38 (51)
Other	1 (1)	0	2 (3)	2 (3)
Does the patient have any of the following comorbidities? n (%)	GP-PRF responses			
	Thailand (N=225)	Brazil (N=225)	Spain (N=225)	Mexico (N=225)
Nasal polyposis	54 (24)	36 (16)	62 (28)	47 (21)
Asthma	143 (64)	71 (32)	117 (52)	79 (35)
Atopic dermatitis	140 (62)	74 (33)	87 (39)	76 (34)
Sinusitis	104 (46)	115 (51)	63 (28)	85 (38)
Otitis media	53 (24)	16 (7)	13 (6)	24 (11)
Conjunctivitis	72 (32)	28 (12)	46 (20)	52 (23)
Other	10 (4)	16 (7)	6 (3)	18 (8)
What symptoms did the patient experience at presentation? Please select all those apply n (%)	GP-PRF responses			
	Thailand (N=225)	Brazil (N=225)	Spain (N=225)	Mexico (N=225)
Nasal stuffiness (congestion)	162 (72)	181 (80)	196 (87)	197 (88)
Sneezing	149 (66)	166 (74)	140 (62)	174 (77)
Runny nose	152 (68)	132 (59)	173 (77)	167 (74)
Itchy nose	142 (63)	153 (68)	130 (58)	131 (58)
Itching in throat and eyes	108 (48)	108 (48)	94 (42)	107 (48)
Headache	58 (26)	88 (39)	68 (30)	77 (34)
Sinus pain	82 (36)	90 (40)	54 (24)	75 (33)
Increased mucus generation in nose and throat	57 (25)	81 (36)	88 (39)	97 (43)
Fatigue	59 (26)	63 (28)	74 (33)	83 (37)
Malaise (general feeling of discomfort)	57 (25)	56 (25)	63 (28)	71 (32)
Sleep loss	43 (19)	65 (29)	52 (23)	57 (25)
Other	3 (1)	2 (1)	0	3 (1)
What symptoms did the patient experience at presentation? Please select all those apply n (%)	Patient survey responses			
	Thailand (N=75)	Brazil (N=75)	Spain (N=75)	Mexico (N=75)
Nasal stuffiness (congestion)	43 (57)	57 (76)	60 (80)	67 (89)
Sneezing	62 (83)	57 (76)	55 (73)	55 (73)
Runny nose	63 (84)	50 (67)	52 (69)	48 (64)
Itchy nose	61 (81)	57 (76)	48 (64)	52 (69)
Itching in throat and eyes	28 (37)	36 (48)	45 (60)	40 (53)
Headache	20 (27)	37 (49)	33 (44)	32 (43)
Sinus pain	29 (39)	24 (32)	23 (31)	18 (24)
Increased mucus generation in nose and throat	26 (35)	34 (45)	44 (59)	35 (47)
Fatigue	20 (27)	13 (17)	11 (15)	12 (16)
Malaise (general feeling of discomfort)	24 (32)	27 (36)	12 (16)	18 (24)
Sleep loss	27 (36)	34 (45)	21 (28)	13 (17)
Other	0	0	0	0
Thinking of the most recent episode of AR at least 3 months ago, which of the following steps did you take to deal with the symptoms? n (%)	Patient survey responses			
	Thailand (N=75)	Brazil (N=75)	Spain (N=75)	Mexico (N=75)

Did not take any action	0	3 (4)	2 (3)	1 (1)
Started non-medicinal treatment (exercise/herbal products/home remedies)	20 (27)	16 (21)	7 (9)	11 (15)
Started medication from my own self-knowledge (self-medication)	29 (39)	24 (32)	11 (15)	14 (19)
Started medication recommended by a local pharmacist	48 (64)	24 (32)	15 (20)	15 (20)
Visited a GP	66 (88)	42 (56)	51 (68)	63 (84)
Visited a specialist (allergist/ENT/pulmonologist)	50 (67)	29 (39)	23 (31)	35 (47)
Other	0	1 (1)	1 (1)	1 (1)

Survey respondents could select more than one answer.

AR, allergic rhinitis; ENT, ear nose and throat; GP, general practitioner; PRF, patient report form.
